# Associations Between Body Image Satisfaction, Body Mass Index, Quality of Life, and Screen Time in Portuguese Students

**DOI:** 10.3390/healthcare13212761

**Published:** 2025-10-30

**Authors:** Jéssica Silva, Joana Serpa, Vanessa Santos, Fernando Vieira, Nuno Casanova, Renata Willig, Fábio Flôres, Priscila Marconcin

**Affiliations:** 1 Instituto Superior de Estudos Interculturais e Transdisciplinares, Instituo Piaget, 2805-059 Almada, Portugal; 2 Insight: Piaget Research Center for Ecological Human Development, Instituto Piaget, 2805-059 Almada, Portugal; joana.serpa@ipiaget.pt (J.S.); nuno.martins@ipiaget.pt (N.C.); renata.willig@ipiaget.pt (R.W.); priscila.marconcin@ipiaget.pt (P.M.); 3 Exercise and Health Laboratory, CIPER, Faculty of Human Kinetics, University of Lisbon, 1495-751 Cruz Quebrada, Portugal; 4 Escola de Ciências Sociais, Universidade de Évora, 7002-554 Évora, Portugal; fabio.flores@uevora.pt; 5 Centro de Investigação em Educação e Psicologia (CIEP), Universidade de Évora, 7002-554 Évora, Portugal; 6 Comprehensive Health Research Centre (CHRC), Universidade de Évora, 7000-645 Évora, Portugal; 7 Faculty of Health Sciences, Universidad Autónoma de Chile, Providencia 7500912, Chile

**Keywords:** adolescents, body image, body mass index, quality of life, screen time

## Abstract

**Background:** This study examined the relationship between screen time, body image satisfaction, body mass index (BMI), and quality of life among children and adolescents from two school clusters in Sesimbra, Portugal. **Methods:** The sample included 80 students aged 10 to 18 years, assessed using validated questionnaires (Collins’ Figure Rating Scale and KIDSCREEN-27). **Results:** Results indicated sex differences in electronic game use, with boys reporting higher usage. A negative association was found between time spent on social networks and perceived autonomy and parent–child relationship quality. A high prevalence of body image dissatisfaction was found, particularly among boys, which was significantly associated with BMI. Regarding quality of life, participants scored lower than the European average in the domains of physical and psychological well-being. **Conclusions:** Findings suggest that screen time, body image, and BMI interdependently affect quality of life, underscoring the need to promote digital literacy, self-esteem, body acceptance, and healthy lifestyles in both school and family settings.

## 1. Introduction

Understanding how children and adolescents perceive their bodies has gained increasing relevance in health, education, and psychology research, given the impact this perception has on physical, psychological, and social well-being. In fact, body image refers to how individuals perceive the shape, size, and appearance of their body, shaped by biological, psychological, social, and cultural factors [1]. Body dissatisfaction occurs when there is a gap between one’s perceived and ideal body image, and it has been consistently linked to adverse outcomes such as low self-esteem, depressive symptoms, eating disorders, and reduced health-related quality of life [1,2,3,4].

Originally proposed by Festinger (1954), Social Comparison Theory posits that individuals evaluate themselves by comparing their attributes with those of others [5]. Recent empirical evidence has extended this framework to the domain of body image, showing that adolescents and young people often assess their physical appearance through comparisons with peers and idealized figures on social media. These upward comparisons have been consistently linked to heightened body dissatisfaction and appearance-related anxiety [6]. With the rise of digital social networks, these opportunities for comparison have multiplied and intensified, as adolescents are daily exposed to edited images that reinforce unrealistic body standards [7,8]. This exposure has been linked to elevated levels of body dissatisfaction, particularly among female adolescents; however, male adolescents also encounter distinct pressures related to muscle development [9]. Hence, adolescence is particularly vulnerable to the influence of idealized beauty standards, as this stage is marked by significant physical, social, cognitive, and emotional transformations. This vulnerability reflects adolescents’ ongoing adjustment to new behaviors and the development of autonomy [10].

Moreover, mass media and social networks expose adolescents to idealized body images that promote unrealistic standards, thereby fostering body dissatisfaction and appearance-related concerns [11,12]. Studies indicate that frequent use of these platforms can lead to unfavorable social comparisons, which amplify feelings of body dissatisfaction among youth [8]. A recent investigation that compiled data from several studies carried out in North America, Europe, Latin America, and Asia indicates that body image dissatisfaction among adolescents aged 12 to 18 varies globally between 25% and 50% in females and between 15% and 30% in males, with differences influenced by the cultural and social contexts of each region [13]. According to the European Union Kids Online study [14], Portugal stood out as one of the countries where the time children and young people spend on digital media has doubled compared to data recorded in 2010. In a related investigation, the Portuguese Psychologists Association [15] found that 64.5% of children and adolescents aged 11 to 17 in Portugal reported using a smartphone as one of their main leisure activities. Another relevant finding is that 36.4% of children and adolescents spend more than two hours per day playing video games, a percentage that rises to 45% among 18-year-olds. According to the Portuguese Society of Neuropediatrics [16], daily screen exposure should be moderated, with a suggested limit of up to one hour per day for children aged 7 to 11, up to two hours for adolescents aged 12 to 15, and up to three hours per day for youth aged 16 to 18.

Another variable associated with body dissatisfaction is sedentary behaviors, which refers to any waking activity that involves very low energy expenditure (≤1.5 METs) and occurs in postures such as sitting, reclining, or lying down, including activities such as watching television or using electronic devices [17]. A longitudinal investigation carried out in China, with 2228 children and adolescents aged 6 to 19, identified a significant correlation between time spent in sedentary screen-based activities and an increase in body mass index (BMI) [18]. Other studies also indicate that high levels of sedentary behavior may contribute to delays in cognitive development and negatively impact academic performance [19]. Moreover, this pattern is related to behavioral difficulties, such as reduced self-esteem, increased anxiety, and deficits in social skills [20]. Sedentary behavior also significantly contributes to increased overweight and body fat during youth [19]. Evidence also highlights an association between sedentary behavior and depressive and anxiety symptoms in adolescents [21]. Low levels of physical activity and the accumulation of sedentary behavior have implications for individual well-being and quality of life [22]. In addition, several studies indicate that overweight and obesity in children and adolescents are associated with a significant decrease in their quality of life [23]. Among youth aged 10 to 18, obesity or overweight is associated with decreased health-related quality of life, especially in the parameters of physical well-being, psychological well-being, and social relationships with peers, compared to the healthy weight group [24]. The health consequences caused by excess weight intensify the importance of conducting regular evaluations of young people [25].

Despite the growing body of international literature linking screen time, body image dissatisfaction, and health-related quality of life (HRQoL), research on these associations remains limited in Southern European contexts, particularly in Portugal. Most existing studies have been conducted in North American or Asian populations [6,12,15], where sociocultural norms regarding body image and digital behavior differ markedly from those of Southern Europe. Recent Portuguese data indicate a marked increase in daily screen exposure among children and adolescents, with approximately two-thirds reporting more than two hours of screen use per day [15], a pattern associated with lower well-being and increased psychological distress [14,26]. However, few studies have examined how screen time interacts with psychosocial factors, such as BMI and body dissatisfaction, to influence perceived quality of life among Portuguese youth.

Additionally, most prior investigations have analyzed these constructs separately, focusing on isolated outcomes (e.g., social media use and body image, or BMI and HRQoL) [8,21,24], rather than exploring their combined and potentially reciprocal effects. Evidence suggests that these variables are interdependent, influencing both health behaviors and emotional development during adolescence, a developmental stage characterized by rapid physical, cognitive, and social transitions [9,27]. Furthermore, cultural gender norms may differentially shape body image perceptions and media use among boys and girls, yet this aspect remains underexplored in Portuguese samples [9,28].

This study aimed to analyze body dissatisfaction in children and adolescents, examine its relationship with BMI, and explore the associations between screen time, perceived autonomy, and health-related quality of life.

Therefore, the present study aims to address this gap by jointly analyzing the associations between body image satisfaction, BMI, screen time, and quality of life among Portuguese children and adolescents. By adopting an integrative, multidimensional approach, this research provides novel empirical evidence and practical implications for promoting digital literacy, self-esteem, and well-being in youth.

## 2. Materials and Methods

### 2.1. Sample

This cross-sectional study included 80 children and adolescents (39 boys and 41 girls) aged 10 to 18 years (mean age 13.5 ± 2.5 years) from two school clusters in the municipality of Sesimbra, Portugal. Participants were selected by convenience sampling between 2024 and 2025. Height and weight were obtained through the FitEscola^®^ program (https://fitescola.dge.mec.pt/home.aspx), conducted by the Physical Education teachers, with a mean height of 1.58 ± 0.10 m, mean weight of 55.1 ± 16.4 kg, and mean BMI of 21.6 ± 4.7 kg/m^2^. Students were distributed across lower secondary education (5th and 6th grades), upper secondary education (7th and 8th grades), and high school (11th grade).

The required sample size was calculated using G*Power v3.1.9.7 (Kiel University, Germany) for a one-way ANOVA with two groups (α = 0.05, power = 0.80, Cohen’s f = 0.455), resulting in a minimum of 80 participants. Inclusion criteria were ages 10–18 years, and the exclusion criterion was the presence of functional limitations or specific needs [29].

### 2.2. Ethics and Consent

The study received approval from the University Ethics Committee (Approval Code: P02-S09-27042022) and adhered to the Declaration of Helsinki (2014). Approval to conduct the survey in schools was obtained from the Directorate-General for Education and registered under number 1696500001 in the School Survey Monitoring database. School boards provided authorization, parents gave informed consent via email, and students provided oral assent before participation.

### 2.3. Instruments

#### 2.3.1. Sociodemographic and Anthropometric Data

Participants provided age, sex, and school grade. Height and weight were measured by physical education teachers according to standardized procedures. BMI was calculated as weight (kg)/height^2^ (m^2^) and classified according to WHO Growth Reference z-scores for ages 5–19 years. Three questions assessed daily time spent on social networks (Instagram, Facebook), electronic games, and video platforms (YouTube, TikTok) with the following options: 0 h, up to 1 h/day, 1–3 h/day, or more than 3 h/day.

#### 2.3.2. Body Image Perception Questionnaire

Body image perception was assessed using the Portuguese version of the Collins Figure Rating Scale for children and adolescents [28]. The scale contains two sets of seven silhouettes (for boys and girls), representing different body weight statuses from 1 (underweight) to 7 (obesity). Participants selected the figure representing their current body image and their ideal body image. Body image dissatisfaction was calculated as the absolute difference between current and ideal figures, with positive values indicating a desire to lose weight, negative values indicating a desire to gain weight, and zero indicating satisfaction.

#### 2.3.3. Quality of Life Questionnaire

Health-related quality of life (HRQoL) was defined as the individual’s perceived physical, psychological, and social well-being in relation to health status and developmental context. HRQoL was assessed using the KIDSCREEN-27 (Portuguese validated version) [30], which covers five domains: physical well-being, psychological well-being, autonomy and parent relations, social support and peers, and school environment. We administered the child/adolescent self-report version appropriate for ages 10–18.

The KIDSCREEN-27 comprises five domains covering distinct aspects of health-related quality of life: (1) Physical well-being (5 items: physical activity, energy, and health perception); (2) Psychological well-being (5 items: positive emotions and satisfaction with life); (3) Autonomy and parent relations (5 items: parental support and perceived independence); (4) Social support and peers (5 items: relationships and social acceptance); and (5) School environment (7 items: learning, concentration, and relationships with teachers). Each item contributes to its respective domain score, which is transformed to a 0–100 scale according to the official scoring manual.

The questionnaire consists of 27 items rated on a five-point Likert scale ranging from “not at all” to “extremely,” assessing the frequency or intensity of experiences during the previous week. Domain scores were computed and standardized according to the official KIDSCREEN-27 manual [31], and transformed to a 0–100 scale, where 0 represents the lowest possible HRQoL and 100 represents the highest. Higher scores indicate better perceived quality of life. No additional grouping or recoding of Likert responses was performed beyond the standard KIDSCREEN scoring procedure.

The Portuguese validated version of the KIDSCREEN-27 has demonstrated good psychometric properties, with Cronbach’s alpha coefficients ranging from 0.78 to 0.84 across domains [30], indicating satisfactory internal consistency and reliability.

### 2.4. Procedures

Parents received the survey link via email to provide informed consent. Questionnaires were completed online by students in classroom sessions under researcher supervision, allowing immediate clarification of any doubts. All instruments were compiled into a single Google Forms questionnaire for ease of administration.

### 2.5. Data Analysis

Data were analyzed using SPSS v29.0 (IBM Corp.). The Kolmogorov–Smirnov test confirmed normal distribution for quantitative variables. Descriptive statistics included means, standard deviations, minimums, and maximums for continuous variables; categorical variables were expressed as frequencies and percentages. Comparisons between groups used chi-square tests for categorical variables and independent samples *t*-tests for continuous variables. Pearson correlations were conducted, interpreted as weak (0.10–0.30), moderate (0.40–0.60), or strong (0.70–1.00) [26]. One-sample *t*-tests compared study data with European reference data from the KIDSCREEN Group Europe study (2006) [32].

## 3. Results

Table 1 presents the distribution of daily screen time across social networks, electronic games/consoles, and video platforms. Statistically significant differences were observed only in electronic gaming, with boys reporting higher usage than girls (*p* < 0.001). As expected, the sample showed a balanced sex distribution and BMI values within the normal range, consistent with a generally healthy youth population.

Table 2 shows the distribution of body image satisfaction, perception, and weight intentions. Significant sex differences were found only for body dissatisfaction, with boys more frequently reporting a desire to gain weight (*p* = 0.042).

Table 3 presents the comparison of quality-of-life domains between sexes. Boys scored significantly higher than girls in physical well-being (*p* = 0.00), while no significant differences were observed in the other domains. Each domain score represents the mean of the items included in that domain, following the HRQoL questionnaire. Participants classified in the unhealthy BMI zone reported significantly lower physical and psychological well-being scores, suggesting that excess weight negatively affects perceived quality of life even in early adolescence.

Table 4 compares the sample’s quality-of-life scores with European data. Significant differences were observed in four of the five domains, with the sample scoring lower in physical and psychological well-being, but higher in autonomy and relationships with parents, and social support and peer relationships; no significant difference was found for the school environment domain.

**Figure 1:**
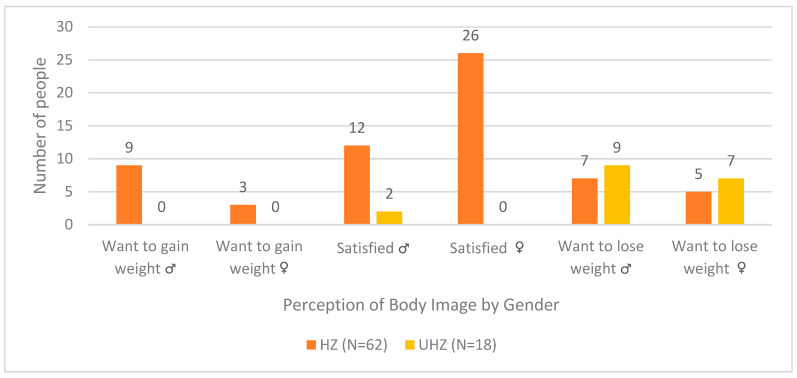
Cross-tabulation between BMI and Body Image. Legend: Participants were classified as ‘Healthy Zone (HZ)’ or ‘Unhealthy Zone (UHZ)’ based on BMI-for-age percentiles according to WHO criteria.

Figure 1 illustrates the correlation between BMI and body image dissatisfaction. A strong positive association was observed (r = 0.73; *p* < 0.001), indicating that participants with BMI outside the healthy zone were more likely to express body dissatisfaction and a desire to lose weight, whereas those within the healthy zone reported higher satisfaction with their body image.

Table 5 shows the mean values of the physical well-being domain, categorized by BMI and sex. There is a tendency for higher physical well-being in individuals with a healthy BMI, with this difference being statistically significant only among girls. These results indicate that higher BMI was associated with greater body dissatisfaction, particularly among girls, supporting previous evidence on sex differences in body perception.

Overall, the data suggest that screen time, body dissatisfaction, and BMI are interrelated factors influencing health-related quality of life among Portuguese adolescents.

## 4. Discussion

This study aimed to advance understanding of body dissatisfaction among Portuguese children and adolescents by examining its associations with BMI, screen time, and quality of life. We hypothesized that higher BMI and longer screen time would be linked to greater body dissatisfaction and lower quality of life, and that girls would report higher levels of dissatisfaction than boys. Overall, our findings partially supported these hypotheses. While higher BMI was associated with increased body dissatisfaction and certain domains of quality of life, girls did not report greater dissatisfaction than boys, as expected. Although this study did not directly analyze the relationship between screen time and body dissatisfaction, previous evidence suggests that this association may be multifactorial, influenced by social comparison, exposure to idealized body images, and individual self-esteem [33,34]. Our findings reinforce the relevance of considering digital habits and media exposure when addressing body image and quality of life among youth.

**Screen time and quality of life.** Most participants reported using social networks and platforms such as YouTube or TikTok between 1 and 3 h per day, while boys reported higher use of electronic games. The present findings are consistent with the existing literature on the subject, which highlights the persistent presence of digital technologies in the routines of young people. Social networks are identified as central tools for communication, entertainment and socialization [35,36,37]. Electronic games were more popular among boys, corroborating previous studies and reflecting both preference differences and the stronger male-oriented marketing of competitive and action games [38].

Moreover, the relationship between screen time, body image, and perceived well-being has been widely discussed in the literature.

Although no statistically significant correlation was observed between time spent on social networks and the Autonomy and Parent Relations domain, a negative trend was noted, suggesting that greater screen exposure may be associated with lower perceived autonomy and less positive relationships with parents. This interpretation aligns with prior research linking higher digital media use to reduced family communication and emotional closeness [39,40].

These findings indicate that greater screen exposure may be associated with less positive family relationships, a pattern not widely reported but partially supported by studies on parental regulation of technology use [41]. Excessive use may reduce face-to-face interactions, weaken family bonds, and foster isolation [42,43]. These findings highlight the need for parental mediation and guidance, as technology alone does not promote autonomy and may hinder the development of emotional independence in adolescence [44]. Body dissatisfaction and sex differences. Regarding body image, a high prevalence of dissatisfaction was found among boys (41% desired a slimmer figure and 23.1% wanted a more muscular silhouette). This may reflect a shift in male body ideals, with increasing emphasis on muscularity and strength rather than thinness [45,46]. Such ideals, promoted through social media, are often difficult to achieve and may contribute to frustration and dissatisfaction among boys [47,48].

Girls, however, showed higher levels of body satisfaction (63.4%), contrary to literature suggesting greater dissatisfaction among females [49]. Greater exposure to diverse body representations in the media may foster more positive perceptions and inclusion [50,51]. Nevertheless, both sexes expressed concerns about body image, with most adolescents perceiving themselves as “neither fat nor thin.” This may indicate some degree of body acceptance or denial of internalized ideals. Emotional support from family, school, and peers can buffer dissatisfaction, promoting stable self-image even under dominant beauty standards [52]. Some adolescents also consciously resist societal ideals, protecting mental health through critical awareness of imposed norms [53,54,55].

**Quality of life domains.** Boys scored significantly higher than girls in physical well-being, a pattern consistent with greater engagement in physical activity and sports [56,57]. Nonetheless, body dissatisfaction was still prevalent among boys, suggesting that good perceived health does not eliminate negative comparisons with idealized standards [46,47]. Regular sports participation may explain their more positive perception of health [58]. In other domains, including psychological well-being, autonomy and parent relationships, social support, and school environment, no sex differences were found. Previous studies suggest that social and school support may act as balancing factors, mitigating disparities [59,60,61].

**BMI and body dissatisfaction.** Adolescents with BMI outside the healthy range reported greater dissatisfaction, mainly in the desire to lose weight. This finding is consistent with prior research showing that elevated BMI is associated with higher dissatisfaction and heightened awareness of discrepancies between actual and ideal body [62,63]. In school’s context, these findings reinforce the importance of creating educational environments that promote body acceptance and discourage weight-related stigma. Adolescents with higher BMI may experience greater pressure to follow socially valued ideals, making them more vulnerable to negative comments or teasing from peers. Such experiences not only intensify body dissatisfaction but can also impair participation in physical education classes, where exposure and comparison are more visible [63]. Schools, therefore, play a crucial role in fostering inclusive practices, such as emphasizing health and well-being over appearance, promoting diverse role models, and integrating activities that build self-esteem and body appreciation. By encouraging positive peer interactions and developing critical media literacy, educators can help students resist unrealistic beauty standards and support healthier relationships with their bodies.

**Implications and limitations.** The findings suggest that while digital technology is a central part of adolescents’ lives, excessive use of social networks may negatively impact autonomy and family relationships. This underscores the importance of parental guidance, critical media education, and structured opportunities for social interaction. Schools, particularly through physical education, can play a key role in promoting healthy lifestyles, balanced technology use, and positive body image. Integrating these themes into curricula and empowering parents and educators may create a supportive environment that minimizes the adverse effects of digital exposure. In addition, the relatively small sample size and the use of European normative data warrant cautious interpretation.

Despite our interesting results, this investigation has some limitations that need to be addressed. The relatively small and homogeneous sample restricts generalizability, while self-reported data may be subject to social desirability bias. Additionally, the cross-sectional design prevents causal inference or assessment of changes over time. Future research should adopt longitudinal designs with larger and more diverse samples to explore the dynamic interactions between body image, BMI, screen time, and quality of life across developmental stages.

## 5. Conclusions

This study contributes to a better understanding of body dissatisfaction and health-related quality of life among Portuguese children and adolescents. Body dissatisfaction was observed in both boys and girls, and greater deviation from BMI reference values was associated with higher levels of dissatisfaction.

No significant associations were found between screen time and body dissatisfaction or overall quality of life. However, a non-significant negative trend was noted between screen time and the Autonomy and Parent Relations domain, suggesting that greater screen exposure may be linked to lower perceived autonomy and less positive family relationships.

These findings highlight the need to monitor digital media use and promote parental mediation strategies to foster healthier digital habits and positive psychosocial development. Future studies should include larger and more diverse samples to further explore the complex relationships between screen time, body image, and well-being in youth.

## Figures and Tables

**Table 1:** Distribution of daily screen time (in hours) on social networks, electronic games/consoles, and video platforms, by sex.

Screen Time in Hours	Social Network			Electronic Gaming			Tik Tok/YouTube		
	Boys (%)	Girls (%)	Total (%)	Boys (%)	Girls (%)	Total (%)	Boys (%)	Girls (%)	Total (%)
0 h	3 (7.7%)	2 (4.9%)	5 (6.3%)	4 (10.3%)	24 (58.5%)	28 (35.0%)	2 (5.1%)	3 (7.3%)	5 (6.3%)
1 h	5 (12.8%)	8 (19.5%)	13 (16.3%)	9 (23.1%)	12 (29.3%)	21 (26.3%)	14 (35.9%)	12 (29.3%)	26 (32.5%)
1 to 3 h	24 (61.5%)	21 (51.2%)	45 (56.3%)	17 (43.6%)	2 (4.9%)	19 (23.8%)	15 (38.5%)	19 (46.3%)	34 (42.5%)
More than 3 h	7 (17.9%)	10 (24.4%)	17 (21.3%)	9 (23.1%)	3 (7.3%)	12 (15.0%)	8 (20.5%)	7 (17.1%)	15 (18.8%)
*p*-value	0.673			0.001 **			0.842		

Significance levels: ** *p* < 0.01.

**Table 2:** Body image assessment, total sample and by sex.

Body Image Variables	Body Image Dissatisfaction			I Think I…			I Would Like of…		
	Boys (%)	Girls (%)	Total (%)	Boys (%)	Girls (%)	Total (%)	Boys (%)	Girls (%)	Total (%)
Want to gain weight	9 (23.1%)	3 (7.3%)	12 (15.0%)	-	-	-	-	-	-
Satisfied	14 (35.9%)	26 (63.4%)	40 (50.0%)	-	-	-	-	-	-
Want to lose weight	16 (41.0%)	12 (29.3%)	28 (35.0%)	-	-	-	-	-	-
Skinny	-	-	-	13 (33.3%)	17 (41.5%)	30 (37.5%)	-	-	-
Neither fat nor thin	-	-	-	19 (48.7%)	19 (46.3%)	38 (47.5%)	-	-	-
Fat	-	-	-	7 (17.9%)	5 (12.2%)	12 (15.0%)	-	-	-
Lose weight	-	-	-	-	-	-	16 (41.0%)	20 (48.8%)	36 (45.0%)
Gain weight	-	-	-	-	-	-	8 (20.5%)	5 (12.2%)	13 (16.3%)
Maintain weight	-	-	-	-	-	-	15 (38.5%)	16 (39.0%)	31 (38.8%)
*p*-value	0.042 *			0.670			0.570		

Significance levels: * *p* < 0.05.

**Table 3:** Quality of life domains and comparison between sex.

Quality of Life Variables	Boys	Girls	Total	t	*p*
	**Mean ± SD**	**Mean ± SD**	**Mean ± SD**		
Physical well-being	67.9 ± 9.5	62.4 ± 8.8	65.1 ± 9.5	−2.67	0.001 **
Psychological well-being	61.2 ± 85.8	60.1 ± 4.4	60.7 ± 5.1	−0.94	0.352
Autonomy and relationship with parents	83.2 ± 11.0	80.5 ± 15.1	82.8 ± 13.4	−1.58	0.123
Social support and peer group	87.0 ± 12.1	82.8 ± 16.9	84.9 ± 14.8	−1.28	0.206
School environment and learning	75.7 ± 13.0	73.3 ± 11.8	74.4 ± 12.4	−0.85	0.407

Significance levels: ** *p* < 0.01; Values are presented as Mean ± standard deviation (SD); The t-statistic (t) and *p*-value (*p*) were used to assess group differences. Scores range from 0 (lowest HRQoL) to 100 (highest HRQoL); higher scores indicate better health-related quality of life.

**Table 4:** Quality of life domains and comparison with European data.

Quality of Life Variables	Total	European Data	*p* Value
Physical well-being	65.1	70.7	0.001 *
Psychological well-being	60.7	76.9	0.001 *
Autonomy and relationship with parents	82.8	74.1	0.001 *
Social support and peer group	84.9	76.9	0.001 *
School environment and learning	74.4	74.1	0.80

Significance levels: * *p* < 0.01.

**Table 5:** Physical Well-being Domain and BMI.

Physical Well-Being	Healthy Zone	Unhealthy Zone
	Mean ± SD	Mean ± SD
Boys	60.5 ± 4.5	58.36 ± 3.2
*p*-value	0.65	
Girls	63.8 ± 8.7	55.71 ± 6.7
*p*-value	0.03 *	
Total	65.8 ± 9.07	62.5 ± 10.8
*p*-value	0.19	

Significance levels: * *p* < 0.05; Values are presented as Mean ± standard deviation (SD).

## Data Availability

The data that support the findings of this study are available from the corresponding author upon reasonable request. Due to privacy restrictions related to minors, the datasets are not publicly available.
